# Next Generation Sequencing in Cytopathology: Focus on Non-Small Cell Lung Cancer

**DOI:** 10.3389/fmed.2021.633923

**Published:** 2021-02-11

**Authors:** Pasquale Pisapia, Francesco Pepe, Antonino Iaccarino, Roberta Sgariglia, Mariantonia Nacchio, Floriana Conticelli, Maria Salatiello, Rossella Tufano, Gianluca Russo, Gianluca Gragnano, Ilaria Girolami, Albino Eccher, Umberto Malapelle, Giancarlo Troncone

**Affiliations:** ^1^Department of Public Health, University of Naples Federico II, Naples, Italy; ^2^CEINGE Biotecnologie Avanzate, Naples, Italy; ^3^Division of Pathology, Central Hospital Bolzano, Bolzano, Italy; ^4^Department of Pathology and Diagnostics, University and Hospital Trust of Verona, Verona, Italy

**Keywords:** molecular cytopathology, cytopathology, NSCLC, fine needle aspiration, cell block, smear, liquid based cytology

## Abstract

Molecular cytopathology is a rapidly evolving field embracing both conventional microscopy and molecular pathology. Its growing popularity stems from the fact that in many types of advanced cancers, including non small cell lung cancer (NSCLC), cytological samples often constitute the only available specimens for morphomolecular analysis. Indeed, non formalin fixed and paraffin embedded (FFPE) cytological samples feature a higher quality of extracted nucleic acids than histological specimens. However, because of the growing complexity of molecular testing, several efforts should be made to validate the analytical performance of the wide array of currently available molecular technologies, including next generation sequencing (NGS). This technology has the terrific advantage of allowing simultaneous detection of scores of predictive biomarkers even in low-input DNA/RNA specimens. Here, we briefly review the role of the modern cytopathologist in the morphomolecular diagnosing of advanced stage NSCLC and the adoption of NGS in conventional cytopreparations (cell blocks, direct smears, and liquid-based cytology) and supernatants.

## Introduction

In the current era of precision medicine, the validation of several predictive biomarkers has dramatically improved the clinical outcomes of advanced cancer patients. For instance, unlike conventional radio-chemotherapeutic agents, tyrosine kinase inhibitors (TKIs) are therapeutically advantageous not only in improving overall patients' survival but also in reducing treatment-associated toxicities (1). However, the molecular heterogeneity of many tumors oftentimes renders some patients unresponsive to these types of treatments. Thus, assessing the molecular status of genomic predictive biomarkers before administration is paramount (2). In this setting, the molecular pathology work-up for advanced-stage non-small cell lung cancer (NSCLC) is a case in point. In fact, various institutions such as the National Comprehensive Cancer Network (NCCN), the College of American Pathologists (CAP), the International Association for the Study of Lung Cancer (IASLC), the Association for Molecular Pathology (AMP), and the American Society of Clinical Oncology (ASCO), strongly recommend the molecular assessment of several biomarkers before administration of TKI treatments. Among these are Epidermal Growth Factor Receptor (*EGFR*), Anaplastic Lymphoma Kinase (*ALK*), ROS Proto-Oncogene 1 Receptor Tyrosine Kinase (*ROS1*), and V-Raf murine sarcoma viral oncogene homolog B (*BRAF*) (3–5). In addition, with the advent of immunotherapy, some studies strongly recommend evaluating the expression level of programmed death-ligand 1 (PD-L1) before the administration of immune-checkpoint inhibitors (ICIs) (6, 7). Despite the rapid increase in the number of clinically relevant biomarkers for advanced-stage NSCLC (8), the scant availability of tissue samples for molecular analysis still poses a major challenge. Undoubtedly, both surgical and biopsy samples still represent the “gold standard” of starting material for molecular purposes, particularly in clinical trials. This is mainly because, unlike cytological preparations, formalin fixed and paraffin embedded (FFPE) histological specimens are characterized by a high quantity of available material for both morphological and molecular evaluation and do not require additional molecular validation. However, in real-world clinical practice obtaining large tissue specimens from advanced-stage NSCLC patients is highly impracticable, if not impossible. Thus, in the vast majority of cases, cytopathologists have to make do with small tissue samples such as endoscopic biopsies and cytological materials (9). Despite this setback, cytological samples are an excellent alternative to histological samples, as evidenced by a flurry of cytological studies. In a previous work, our research team demonstrated that cytological specimens display a better quality of nucleic acids despite featuring reduced starting input (10). In fact, as opposed to histological samples, whose long fixation time may give rise to C > T artifacts, cytological samples do not suffer from formalin-based fixatives or long fixation time periods, thereby yielding far fewer false positive molecular results (11). Moreover, the valid option for cytopathologists to use cell block (CB) preparations for banked tissue archives enables them to retain the cellular morphology of unique and unrepeatable diagnostic slides for molecular analysis (10).

Interestingly, typical cytopreparations (CBs, smears, and liquid based cytology) are not the only alternatives to histological samples. Indeed, several studies have investigated the possibility of using supernatants for molecular analysis. Until recently, supernatants were typically discarded after cytological preparations. Nowadays, instead, they are being used as a valid source of tumoral nucleic acids for molecular analysis, enabling preservation of diagnostic slides (12).

**Figure 1:**
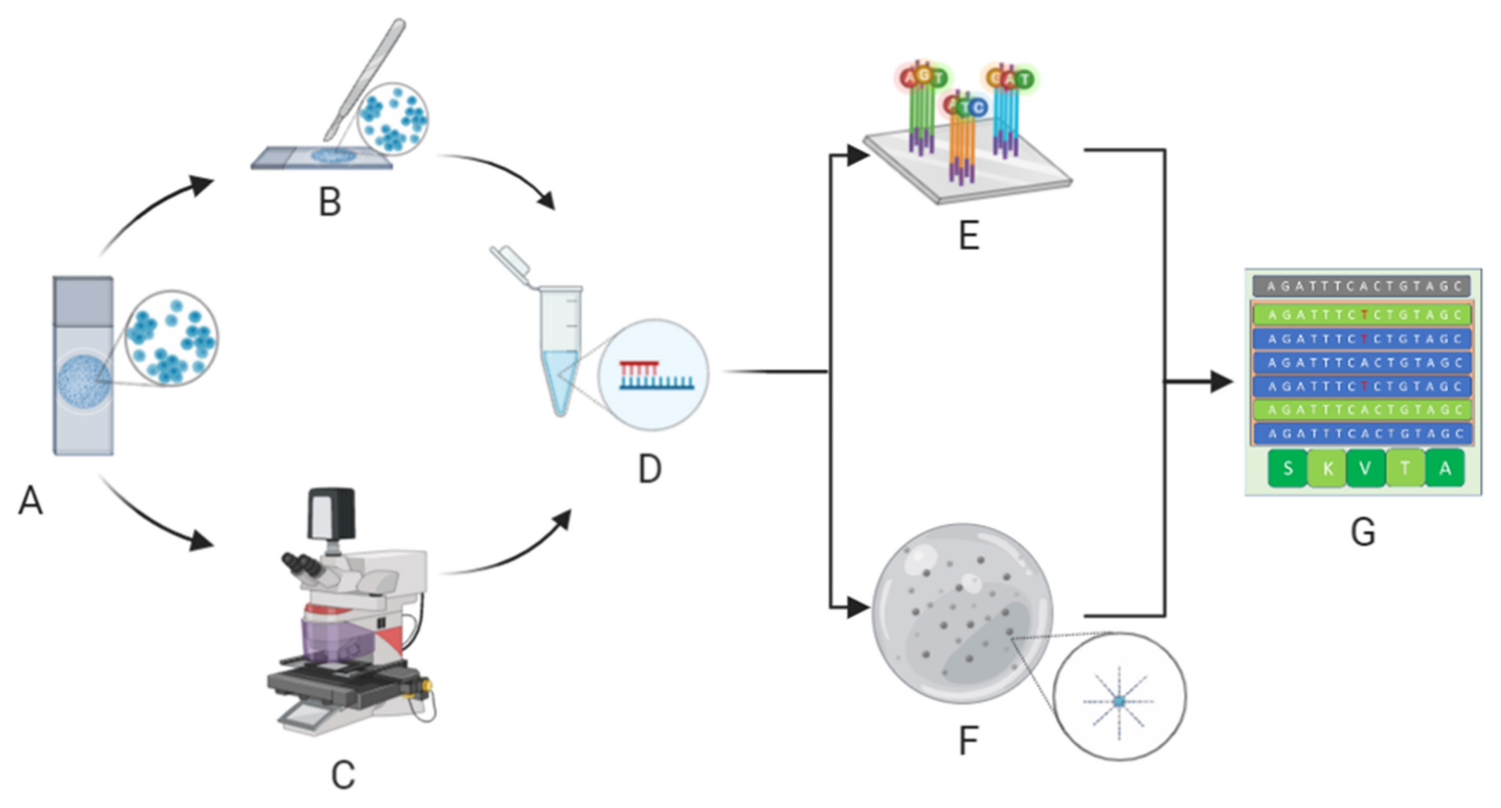
Molecular cytopathology workflow. After microscopy, evaluation of neoplastic cell content whilst avoiding contaminants **(A)** is carried out by manual dissection of cytological samples **(B)** or, in particularly difficult cases, laser microdissection **(C)** before extraction of nucleic acids **(D)**. Extracted DNA/RNA is adequately adopted for next generation sequencing analysis, specifically hybrid-capture-based **(E)** or amplicon-based **(F)** platforms, to define the molecular status of clinically relevant biomarkers **(G)**. (Credit: Created with Biorender.com).

Since different types of cytopreparations are suitable for molecular analysis (13), the validation process of these samples is key. For example, the updated CAP/IASLC/AMP guideline recommends the adoption of cytological smears for molecular analysis in advanced-stage NSCLC patients (5, 14). Moreover, our research team and others (15) have demonstrated that the hurdle of low quantities of DNA/RNA yield from cytological samples may be successfully overcome by implementing next generation sequencing (NGS) technologies (Figure 1). Indeed, NGS is an amazingly versatile tool able to analyze different biomarkers for different patients simultaneously (16).

Here, we briefly review the role of modern cytopathologists in the treatment decision making algorithm of advanced-stage NSCLC patients. Further, we summarize the latest literature on the application of NGS not only to conventional cytopreparations (CBs, direct smears, and liquid-based cytology) but also to supernatants.

## The role of Modern Cytopathologists

Over the last decade, cytopathology practice has undergone a paradigm shift in the rapidly evolving field of molecular cytopathology. In this fast-changing scenario, modern cytopathologists have made great strides in the “world” of molecular testing, thus ensuring comprehensive management of advanced-stage cancer patients in modern multidisciplinary team (MDT) settings (17). In this context, the role of traditional histology and cytology has been enriched with the advent of molecular analysis. In this scenario, both cytopathologists and histopathologists can no longer work separately from other healthcare professionals. Indeed, being the “custodians” of tissue specimens, modern cytopathologists must dynamically interact with other healthcare figures to ensure adequate management of samples suitable for NGS analysis. By doing so, they can support clinicians in treatment decisions by providing accurate predictive outcomes in patients with advanced-stage NSCLC (17). Thus, owing to their morphological and molecular skills, modern molecular cytopathologists act as a liaison between clinicians and molecular biologists, both involved in the molecular predictive pathology workflows (10).

Overall, molecular cytopathologists are pivotal in the pre-analytical evaluation of cytological samples for molecular analysis (18). In this setting, their role is to review the adequacy of retrieved cytological material in terms of neoplastic cell content and the presence of contaminants, which may either lead to false negative results (e.g., non-neoplastic cells or necrosis) or inhibit the amplification process (e.g., mucus) (19). On the other hand, when samples are deemed inadequate for molecular analysis, cytopathologists must revoke clinician requests for the analysis, making sure to explain the reason for its rejection in a detailed written report (10). Another possible scenario involving molecular cytopathologists is the possibility of requesting biomarker assessment immediately after morphological diagnosis without the need for a specific request from clinicians (20). The advantage of this possibility, called “reflex testing,” is that it reduces the lapse of time between the morphological diagnosis and the administration of targeted treatment. Despite being highly advantageous, reflex testing is however limited by the high costs associated with molecular analyses, which are not always sustainable by molecular predictive pathology laboratories, compared with on-demand testing (13).

## An Overview of NGS Application on NSCLC Cytological Samples

In the era of precision medicine, molecular analysis on cytological samples has acquired a relevant role in the management of advanced stage NSCLC patients (21). Different molecular assays are currently available (Table 1). Among these, NGS represents a fascinating tool able to analyze different biomarkers for different patients simultaneously, even when applied to cytological samples with low DNA/RNA yield (22). Generally, all NGS technologies share three sequencing approaches, namely, synthesis, hybridization, and ligation (23). In addition, all NGS workflows entail four identical steps: (1) library preparation; (2) clonal amplification of single generated fragments; (3) massive parallel sequencing, and (4) data analysis (22). In particular, genomic libraries comprise “captured” DNA fragments ready for sequencing. Fragment capture can be achieved with two platforms: The Ion Torrent technology (Thermo Fisher Scientific, Waltham, MA), and Illumina technology (Illumina, San Diego, CA). The former adopts polymerase chain reaction (PCR) multiple primer pairs to make targeted capture of DNA/RNA fragments (16). Conversely, the latter employs a hybridization system (24). Clonal amplification enables the clonal expansion of single generated fragments in hundreds of thousands of copies. It is achieved through emulsion PCR on beads by the Ion Torrent platform (25), or through emulsion PCR on a solid support on a flat glass microfluidic channel (flow cell) by the Illumina platform (26) Massive parallel sequencing is achieved on semiconductor chips by the Ion Torrent platform. These chips are able to detect changes in pH elicited by the release of a hydrogen ion (H+) within each well, after non-labeled nucleotide incorporation by DNA polymerase (27). Conversely, massive parallel sequencing is achieved with nucleotides labeled with reversible dye terminators by the Illumina platform (26). Finally, data analysis is achieved by specific bioinformatics pipelines (28).

**Table 1:** Overview of currently available molecular assays.

**Assay**	**LOD (%)**	**Reference range**
FISH	–	Only covered alterations (probe based)
Sanger sequencing	10.00–20.00	All alterations within the analyzed gene regions
RT-PCR	1.00–5.00	Only covered alterations (probe based)
dPCR	0.10–1.00	Only covered alterations (probe based)
Multiplex digital color-coded barcode	5.00–10.0	All alterations within the analyzed gene regions
NGS	0.01–5.00	All alterations within the analyzed gene regions

*dPCR, digital polymerase chain reaction; FISH, fluorescent in situ hybridization; LOD, limit of detection; NGS, next generation sequencing; RT-PCR, real time polymerase chain reaction*.

## Cytological Specimen Cellularity for NGS Analysis and Enrichment Strategies

Adequacy of cytological samples for NGS analysis is assessed according to tumor cellularity and viability. Both factors may, however, vary depending on the analytical sensitivity of the platform used (29). As mentioned above, limited tissue availability is an important issue to consider. Although there is still a lack of a standardized cut-off tumor cell requirement, some authors suggest that tumor cellularity for NGS analysis should be more than two-fold the limit of detection recommended by the molecular assay (30). Regarding tumor cell viability, care should be taken to discriminate viable tumor cells against confounding components, including mucus, necrosis, melanin, and non-neoplastic cells (10, 31).

As a general rule, to assess whether a sample is appropriate for NGS analysis, cytopathologists should ascertain that the fraction of tumor cells is equal to or higher than 20% (31). However, in samples showing high global cellularity with low tumor fraction content, cytopathologists should carefully delineate the area with the highest tumor cell content to avoid contamination with non-neoplastic contaminants (10, 31). Conversely, in samples showing low cellularity with high tumor fraction, cytopathologists should adopt additional CB sections, smears, or cytospins (10, 31).

## Cell Block Preparations

CBs could be considered “hybrid” versions of cytological and histological samples. Several studies have highlighted their utility and, under certain circumstances, their advantages over other types of preparations. For instance, a major benefit of using CBs instead of other cytological preparations is the possibility of performing ancillary studies, including molecular testing, without the need of additional validation. This aspect is important as it enables cytopathologists to preserve diagnostic slides (32). Accordingly, the first edition of the CAP/IASLC/AMP molecular testing guidelines strongly recommends adopting CBs rather than smear preparations for molecular purposes in advanced-stage NSCLC patients (14). The usefulness of CBs preparations in NSCLC has been widely demonstrated. For instance, a few years ago, Hwang et al. successfully analyzed 29 CB specimens, including 15 NSCLC samples by using a hybrid-capture NGS approach with a broad gene panel (309 cancer-related genes) (33). Similarly, our cytology laboratory has successfully demonstrated the usefulness of CB samples in assessing clinically relevant biomarkers in advanced-stage NSCLC patients (34). Indeed, our custom NGS narrow panel (SiRe®) successfully analyzed the vast majority of CBs (86.7%, 39/45). Overall, we were able to detect 15.4% *EGFR*, 33.3% Kirsten Rat Sarcoma Viral Oncogene Homolog (*KRAS*), and 7.7% *BRAF* mutated cases (34). Regarding NGS run parameters, we found no statistically significant differences between smears and CBs (34). Consistently, Zhang et al. performed NGS analysis on 16 CB samples obtained from pleural effusions or fine needle aspirations performed on primitive or metastatic lung adenocarcinoma lesions (35). By adopting a six gene panel, they obtained an overall high success rate of 93.8% (15/16). In particular, a total of 11 gene mutations [nine *EGFR*, one *KRAS*, and one Phosphatidylinositol-4,5-Bisphosphate 3-Kinase Catalytic Subunit Alpha (*PIK3CA*) mutation] were detected (35). Notably, in a case study of a patient with lung cancer that had metastasized to the phalanx, Clery et al. adequately performed NGS analysis on CBs prepared from the metastasized phalanx fine needle aspiration (FNA). The authors demonstrated the usefulness of CBs not only for ancillary techniques (e.g., immunocytochemistry), but also for the molecular assessment of the clinically relevant biomarkers ordered by the patient's oncologist (the patient harbored an “uncommon” *EGFR* exon 20 p.S768_D760DUP). Strikingly, all testing was performed without sacrificing any precious diagnostic material (36). Taken together, this case study indicates that the CB preparations represent a valuable option even for the morphomolecular evaluation of rare lung cancer metastases. However, in choosing among the different types of cytopreparations, molecular cytopathologists should take into account that CBs, like histological preparations, require formalin fixation, which may give rise to confounding artifacts and loss of nucleic acid yield. Results are summarized in Table 2.

**Table 2:** Summary of studies adopting next generation sequencing on cytological samples.

**References**	**Platform**	**Panel**	**Sample type**	**Total number of samples**	**Adequate samples (%)**
Pepe et al. (34)	Ion S5 System™ (Thermo Fisher Scientifics)	Custom Panel (7 genes)	Direct smear cell block	135	125 (92.6)
				45	39 (86.7)
Zhang et al. (35)	Ion PGM™ (Thermo Fisher Scientifics)	NextDaySeq Lung panel (7 genes)	Cell block	16	15 (93.8)
Karnes et al. (37)	HiSeq 2000 (Illumina)	WUCaMP 27 panel (27 genes)	Direct smear	5	5 (100.0)
Treece et al. (38)	MiSeq (Illumina)	Custom Panel (26 genes)	Direct smear	9	9 (100.0)
Velizheva et al. (39)	Ion PGM™ (Thermo Fisher Scientifics)	Oncomine DNA panel for Solid Tumors and Fusion Transcripts (26 genes)	Direct smear	8	7 (87.5, RNA-based)
					8 (100.0, DNA-based)
Fielding et al. (40)	MiSeq (Illumina)	TruSeq Amplicon Cancer Panel (48 genes)	Direct smear and cell block	67	62 (92.5)
Reynolds et al. (41)	Ion PGM™ (Thermo Fisher Scientifics)	Ion AmpliSeq Cancer Hotspot Panel (50 genes)	Liquid based cytology	49	37 (77.5)
Roy-Chowdhuri et al. (12)	Ion Proton (Thermo Fisher Scientifics)	Ion AmpliSeq Cancer Hotspot Panel v2 (50 genes)	Supernatant	35	35 (100.0)
Guibert et al. (42)	NextSeq (Illumina); digital droplet PCR	IAseq Targeted ActionableSolid Tumor Panel (20 genes)	Supernatant	17	17 (100.0)
Janaki et al. (43)	Ion PGM™ (Thermo Fisher Scientifics)	Solid Tumor Focus Assay (69 genes)	Supernatant	30	30 (100.0)
Hannigan et al. (44)	Ion Proton (Thermo Fisher Scientifics)	Ion AmpliSeq Cancer Hotspot Panel v2 (50 genes)	Supernatant	116	104 (89.7)

## Direct Smears

Being either air dried or ethanol fixed, direct smears provide a higher quality of extracted nucleic acids compared with formalin fixed samples, like histological specimens and CBs (10). However, smears do present a few setbacks. For instance, as opposed to other cytopreparations, they require careful validation for any given molecular approach (45, 46). In addition, their uniqueness may substantially limit the adoption of morphological diagnostic slides for molecular purposes (10). On the other hand, it has been widely demonstrated that direct smears, as well as FFPE specimens, may be suitable for complete and precise NGS analysis. For instance, the updated CAP/IASLC/AMP molecular testing guideline strongly suggests the adoption of smears as suitable starting material for molecular testing in advanced-stage NSCLC patients (5). Consistently, in a limited sample set (*n* = 5 direct smears either air-dried, methanol-fixed, or ethanol-fixed, and paired FFPE samples), Karnes et al. demonstrated the suitability of FNA lung adenocarcinoma smears for NGS analysis (37). Indeed, despite containing lower amounts of input DNA, the authors showed that direct smears displayed overlapping results with FFPE specimens in terms of sequencing run parameters and single nucleotide variant detection (overall concordance of 99.5% between Diff Quik, Papanicolaou stained smears, and histological samples) (37). Continuing in this line of research, a few years later, Treece et al. retrieved ten archival Diff Quik stained smears of nine patients previously tested for *EGFR* and *KRAS* mutational status with pyrosequencing and Sanger sequencing (38). Impressively, their NGS analysis confirmed the presence, or absence, of *EGFR* and *KRAS* mutations in all instances. In addition, it also identified several other mutations that had previously been missed by more conventional methodologies probably because of their limited reference range (38).

Besides being suitable for DNA-based NGS analysis, direct smears may also be appropriate for RNA-based NGS testing. In this regard, Velizheva et al. obtained 100.0 and 92.0% successful rates when implementing DNA- and RNA-based NGS approaches (39). In particular, high levels of sensitivity (100% for both DNA and RNA) and specificity (96.0 and 100.0% for DNA and RNA, respectively) were reported (39). In accordance with these findings, Fielding et al. achieved high concordance rates of detected mutations between CBs and matched smears obtained from endobronchial ultrasound-guided transbronchial needle aspiration. In addition, the authors showed that DNA extracted from smears and subjected to NGS yielded higher rates of mutations than did DNA from CBs. Based on these data, the authors finally concluded that smears should be employed in cytology laboratories as the primary source for NGS analysis to spare CB slides for diagnostic analyses, including immunocytochemistry for ALK, ROS1, and PD-L1 (40). This hypothesis was also empirically supported by our research team. Indeed, after subjecting numerous NSCLC smears (*n* = 135) to NGS analysis to assess the molecular status of clinically relevant biomarkers, we observed that only a few smears (7.4%, 10/135) generated inadequate libraries. Indeed, 20.0, 26.4, 4.0, and 0.8% of the samples revealed *EGFR, KRAS, BRAF*, and Neuroblastoma RAS Viral (V-Ras) Oncogene Homolog (*NRAS*) gene mutations, respectively (34). Once again, these data suggest that smears, like CBs, can be successfully applied to NGS to identify predictive biomarkers in lung cancer patients. Results are summarized in Table 2.

## Liquid Based Cytology

Liquid based cytology (LBC) represents a valuable alternative to more conventional cytopreparations to avoid inadequate management of aspirated material by untrained clinicians (47). Indeed, whereas conventional preparations may be compromised by the presence of confounding material, this technique enables cytopathologists to differentiate adequate from inadequate material. The reason is that the aspirated material can be quickly collected and preserved in alcohol-based media (48). Above all, it offers the potential to retrieve residual material for molecular testing. In this regard, Reynolds et al. demonstrated the suitability of residual cell pellets from LBC preparations for NGS analysis. In particular, they adopted AmpliSeq Cancer Hotspot Panel v2.0 on Ion Torrent Personal Genome Machine platform (Thermo Fisher Scientific). Remarkably, by applying this platform to 20 archival LBC cell pellet materials, the authors successfully detected not only 12 *EGFR* mutations, but also *KRAS, NRAS*, MET Proto-Oncogene, Receptor Tyrosine Kinase (*MET*), Erb-B2 Receptor Tyrosine Kinase 2 (*ERBB2*), and Phosphatidylinositol-4,5-Bisphosphate 3-Kinase Catalytic Subunit Alpha (*PIK3CA*) gene alterations. Of note, real-time polymerase chain reaction (RT-PCR) confirmed all *EGFR* mutations, whereas the MiSeq platform (MiSeq, Illumina, San Diego, CA) confirmed the other detected alterations (41).

Another major advantage of this approach is that LBC samples can be used to build quality controls for different NGS platforms to standardize molecular procedures on cytological specimens. To this end, the Molecular Cytopathology Meeting Group, which involves highly specialized molecular cytopathogists from all over the world, developed artificial genomic reference standards in cytocentrifuge/cytospin format to validate the feasibility of using NGS on routine cytopreparations (49, 50). For example, in two recent studies, cell lines were genetically engineered to harbor clinically relevant “common” and “uncommon” mutations in solid tumors, including NSCLC, at different dilution points. These cell lines were then processed and analyzed by all participating laboratories according to their routine staining protocols and in-house NGS platforms, respectively. Notably, all laboratories were able to detect almost all targeted mutations within the cut off thresholds adopted for clinical relevance on tissue specimens (10 and 5%). In addition, the challenge of detecting low frequency mutations (e.g., 1%) was circumvented by resorting to visual inspection to avoid missing potentially relevant gene alterations (49, 50). Results are summarized in Table 2.

## Supernatants

Discarded supernatant fluids of FNA needle rinses after centrifugation and cell pelleting may be a valuable source of high quality nucleic acids for molecular purposes in lung cancer patients (51). This strategy may be useful to avoid sacrificing precious and unique diagnostic cytological slides needed for molecular analysis. In addition, it allows cytopathologists to carry out molecular predictive analyses even when cytology slides are deemed inadequate or insufficient (51). In effect, several studies have widely demonstrated the potential use of supernatants for NGS analysis. For example, in three lung adenocarcinoma samples, with inadequate cytological tissue material for molecular analysis, Roy-Chowdhuri et al. successfully performed NGS analysis on DNA extracted from discarded supernatant from centrifuged lung nodule FNA needle rinses collected in RPMI medium (12). Interestingly, the authors were able to detect clinically relevant mutations in all supernatant FNA samples (*n* = 1 *EGFR* exon 21 p.L858R, *n* = 1 *EGFR* exon 19 p.E746_A750del plus *EGFR* exon 20 p.T790M, and *n* = 1 *KRAS* exon 2 p.G12V) (12). Similarly, Guibert et al. ably analyzed DNA extracted from supernatant FNAs of 12 lung adenocarcinoma patients (*n* = 6 newly diagnosed and *n* = 6 with lung cancer and acquired resistance to TKIs) (42). Impressively, in such a newly diagnosed setting, the sequencing results from supernatant specimens were completely concordant with those from tissue specimens. Of note, in one instance the molecular analysis was successfully carried out even in a case with inadequate tissue sample. Regarding *EGFR* exon 20 p.T790M point mutation, a concordance of 100.0% was reported between the tissue and supernatant samples (42). Likewise, absolute concordance (100.0%) between tissue and supernatant samples has also been reported by Janaki et al. in *n* = 30 endobronchial FNAs (43). The possibility of applying NGS to tumor-derived DNA from supernatant has also been substantiated by Hannigan et al. (44). Overall, the authors successfully analyzed 104 (89.7%) out of 116 samples and detected a total of 155 somatic mutations in 85 (81.7%) samples. In one instance, an *EGFR* exon 20 p.T790M point mutation was detected in the supernatant sample but not in the corresponding tissue specimen. Of note, the mutation detected in the supernatant was further confirmed by digital droplet PCR (44). Results are summarized in Table 2.

## Future Directions

Precision medicine, accompanied by novel targeted treatments, has significantly modified the way advanced-stage NSCLC is treated. In this setting, modern molecular pathology, based on traditional morphological pathology, has a pivotal role in the diagnostic algorithm and subsequent treatment decision making for patients with advanced-stage NSCLC. It is in this context that traditional morphology merges with molecular cytopathology. To date, modern cytopathologists and histopathologists are not only the “custodians” of tissue specimens, but also major players in the management and prioritization of tumor material destined for molecular testing (17). Generally, molecular cytopathologists have rightfully earned their place at the table of modern multidisciplinary teams (17). However, the ever-increasing molecular knowledge of diseases, alongside novel sequencing technologies, such as NGS, is deeply changing the way cytopathology is practiced. In this setting, it is pivotal for the novel generation of cytopathologists to receive adequate training in the intricate realm of molecular techniques (52, 53). Indeed, despite requiring careful validation, cytological samples have proven suitable for NGS analysis when DNA-based biomarkers are taken into account and histological tissues are unattainable. Ample evidence demonstrates that NGS applied to cytological samples may be useful to detect not only point mutations and deletions but also RNA-based clinical relevant biomarkers, such as gene rearrangements (54, 55). Using cytological samples, several studies have indeed obtained high quality RNA suitable for the NGS analysis of gene rearrangements (56, 57).

**Figure 2:**
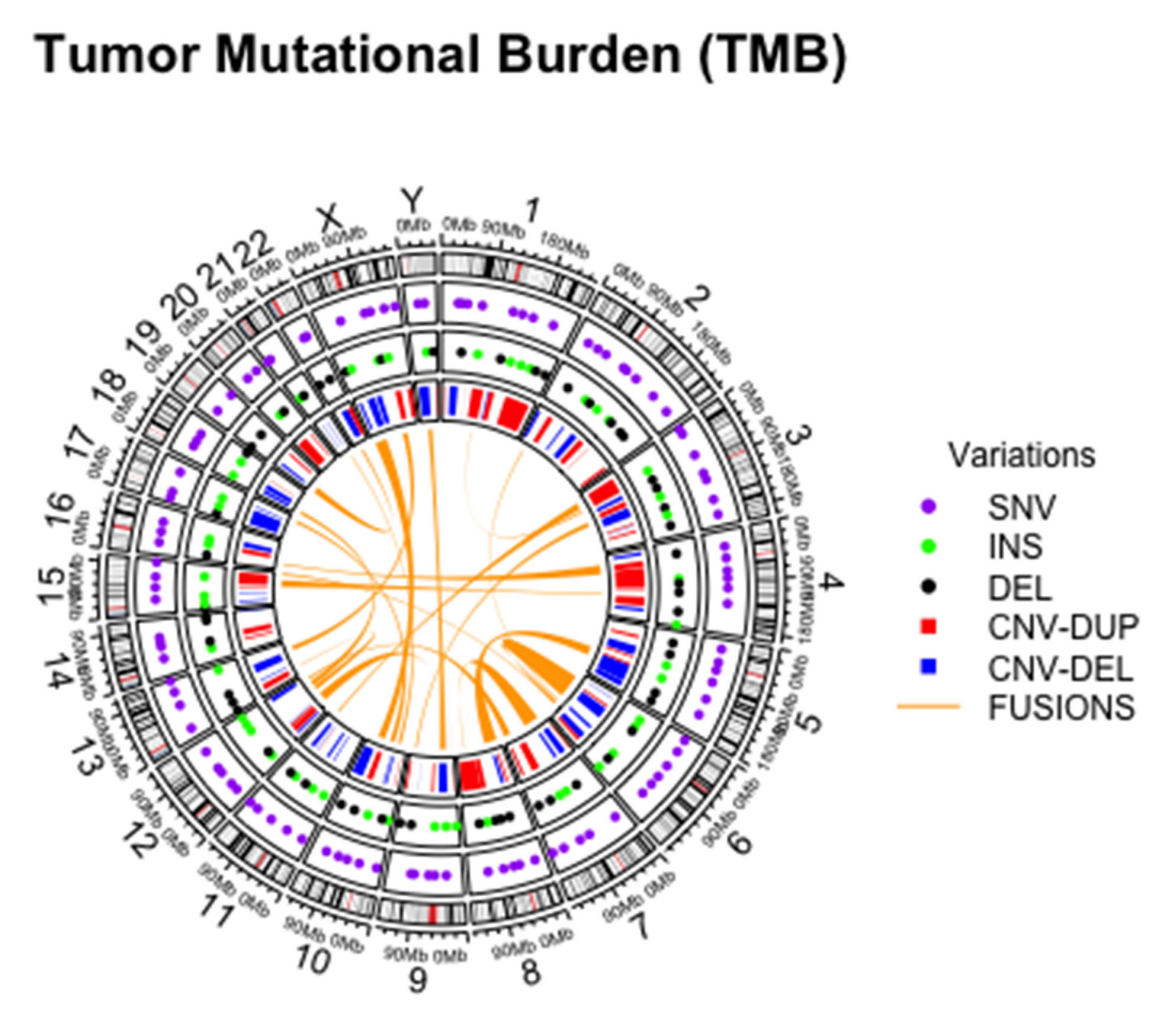
Graphical representation of different alterations within tumor mutational burden analysis on cytological sample.

In addition, tumor mutational burden (TMB) has emerged as a highly complex and independent predictor of response to immunotherapy. Briefly, TMB is the total number of somatic, coding, base substitutions, and short insertions/deletions (indels) per tumor genome (Figure 2) (58, 59). Considering its high prevalence in solid tumors, molecular cytopathologists should also be ready to face the challenge of evaluating this complex biomarker in advanced NSCLC patients (60, 61).

In conclusion, this review briefly evidenced the active role of the modern cytopathologists in ensuring successful NGS analysis of cytological samples and in contributing to treatment decision-making in advanced-stage NSCLC. Moreover, it has highlighted the value of cytological preparations as starting material for NGS analysis. Indeed, not requiring long fixation time and formalin-based fixatives, some of these preparations generate even better DNA/RNA yields than conventional samples. Moreover, the use of supernatants for detection of clinically relevant driver genes of NSCLC, or other types of advanced cancers, enables cytopathologists to preserve unique and precious cytological slide material indispensable for molecular analysis. Therefore, given the complexity of this ever-evolving clinical scenario, we recommend continuous and adequate training programs for modern molecular cytopathologists.

## Author Contributions

PP and GT: conceptualization. PP, UM, and GT: writing—original draft preparation. GT: supervision and project administration. All authors: methodology, software, validation, formal analysis, investigation, resources, data curation, writing—review and editing, and visualization.

## Conflict of Interest

UM reports personal fees (as speaker bureau or advisor) from Boehringer Ingelheim, AstraZeneca, Roche, MSD, Amgen, and Merck, unrelated to the current work. GT reports personal fees (as speaker bureau or advisor) from Roche, MSD, Pfizer, and Bayer, unrelated to the current work. The remaining authors declare that the research was conducted in the absence of any commercial or financial relationships that could be construed as a potential conflict of interest.
